# Pushing the Eenvelope in Battery Estimation Algorithms

**DOI:** 10.1016/j.isci.2020.101847

**Published:** 2020-11-23

**Authors:** Anirudh Allam, Edoardo Catenaro, Simona Onori

**Affiliations:** 1 Department of Energy Resources Engineering, Stanford University, Stanford, CA 94305, USA

**Keywords:** Engineering, Energy Engineering, Energy Storage, Energy Systems, Energy Materials

## Abstract

Accurate estimation of lithium-ion battery health will (a) improve the performance and lifespan of battery packs in electric vehicles, spurring higher adoption rates, (b) determine the actual extent of battery degradation during usage, enabling a health-conscious control, and (c) assess the available battery life upon retiring of the vehicle to re-purpose the batteries for “second-use” applications. In this paper, the real-time validation of an advanced battery health estimation algorithm is demonstrated via electrochemistry, control theory, and battery-in-the-loop (BIL) experiments. The algorithm is an adaptive interconnected sliding mode observer, based on a battery electrochemical model, which simultaneously estimates the critical variables such as the state of charge (SOC) and state of health (SOH). The BIL experimental results demonstrate that the SOC/SOH estimates from the observer converge to an error of 2% with respect to their true values, in the face of incorrect initialization and sensor signal corruption.

## Highlights

• An electrochemical adaptive interconnected observer is used to estimate cell SOC/SOH.
• A description for setting up state-of-the-art BIL experiments is presented.
• A SOC/SOH estimation error of 2% with respect to the measured true values is reported.
• The observer is robust to noise, quantization errors, and initialization errors.

## Graphical Abstract

**Figure:**
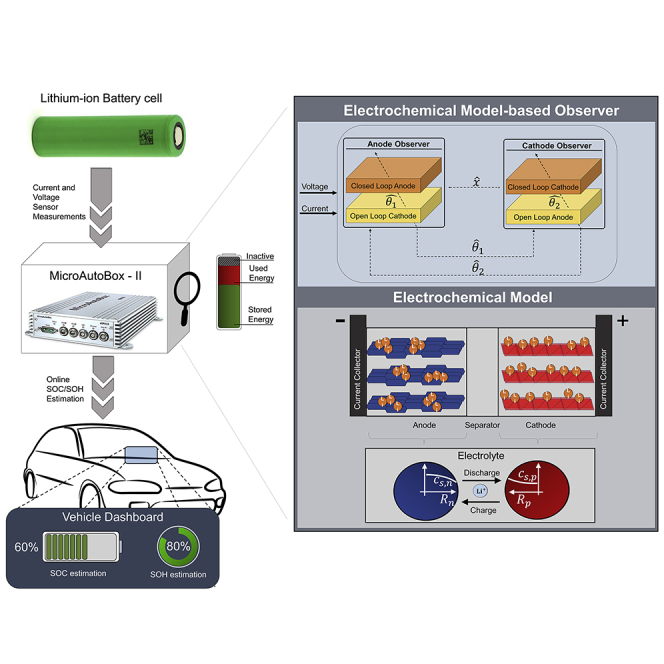

## Introduction

The expanding global electric vehicle market is an indication of a conscious effort by civilization to reduce the reliance on fossil fuels and steadily replace it with a more environment-friendly energy storage and conversion system. Lithium-ion batteries (LIBs) are electrochemical energy storage systems that have found themselves to be the preferred choice for the electrification of the transportation sector and being considered as a storage solution in the renewable energy sector owing to their superior specific energy and power density. Despite these benefits, LIBs are known to be susceptible to abuse (such as thermal runaway (Wang et al., 2012)) due to complex degradation mechanisms, which may lead to safety and reliability issues. For this reason, an LIB system is accompanied with a battery management system (BMS) with the main objective of ensuring its safety, performance, and reliability. The Battery Management System (BMS) is tasked with the responsibility of monitoring the critical battery internal variables that represent the current state of charge (SOC) and state of health (SOH) (Rahimi-Eichi et al., 2013) and uses that information for maintaining conditions conducive for a longer battery lifespan. Typically, these critical variables are not available for measurement via sensors, and hence, the BMS has to “estimate” these variables from available battery current, voltage, and temperature data.

### Motivation

Model-based observers are widely researched in the literature for the combined estimation of SOC and SOH. These model-based estimation algorithms can be broadly classified into two main groups, based on the type of battery model they use: equivalent circuit model and electrochemical model. While the estimation algorithms based on equivalent circuit models (Chen et al., 2014; Kim, 2006) are computationally inexpensive and easy to implement, it needs to be pointed out that they do no offer physical insight into battery's internal dynamics, and a large amount of experimental effort goes into accurately calibrating these lumped-parameter models for different operating conditions. On the other hand, electrochemical models are characterized by lithium ion transport mechanisms, and hence, using such detailed physics-based models for estimation lends itself well to accurately monitoring the internal battery variables such as lithium concentration (Dey et al., 2015; Moura et al., 2017; Allam and Onori, 2018, 2020b) and by extension the SOC and SOH. Naturally, electrochemical model-based estimation is more powerful and can also provide key physical insights to the BMS to take precise control decisions to improve safety and lifespan. However, the bottleneck with this method is that it is computationally expensive since the model is described by a system of partial differential equations (PDEs).

Consequently, discussions on the real-time performance of these electrochemical model-based observers have been lacking in the literature. Further, there is no documented evidence of this method being implemented on real hardware. Therefore, one of the key steps in demonstrating the strength of the class of electrochemical model-based observers is to show that they can be robust against challenges introduced during real-time implementation, such as noisy signals and computational constraints. Hence, it is imperative to test such an observer in real time on a physical hardware to gauge its performance holistically.

To that end, this work aims to present a detailed experimental framework to enable the real-time validation of a reduced-order electrochemical model-based observer via battery-in-the-loop (BIL) experiments by using state-of-the-art equipment.

### Battery-in-the-Loop Experiments

Modern day electric vehicles are complex systems consisting of numerous electronic control units (ECUs), such as the BMS ECU, that have specific functionalities, and they also interact with each other. Each ECU may have high development cost and time associated with it. The primary objective of automotive manufacturers is to reduce development and testing time of ECUs, testing costs, and ensure safety while fault testing and validating these subsystems. For that reason, the ECU development workflow involves different simulation/testing stages such as

1. Model-in-the-loop (MIL): The MIL test is conducted at the initial stages of the V-model workflow, wherein the plant model to be controlled and the controller (ECU) model are in a simulation environment, and no physical hardware components are involved.
2. Software-in-the-loop (SIL): The SIL stage involves the code generation of the controller (ECU) model, which is tested with the plant model in a simulation environment with no physical hardware components.
3. Hardware-in-the-loop (HIL): The HIL stage involves code generation of both, the plant and controller models. The plant code is downloaded to a real physical hardware simulator and the controller code is hosted on an embedded controller. Physical hardware components and connections such as sensors, actuators, physical wiring interconnections are a part of this test.

Each of the above tests is conducted at different stages of the ECU development workflow to gain confidence in the performance of the controller algorithm, both in simulation and hardware environment.

**Figure 1:**
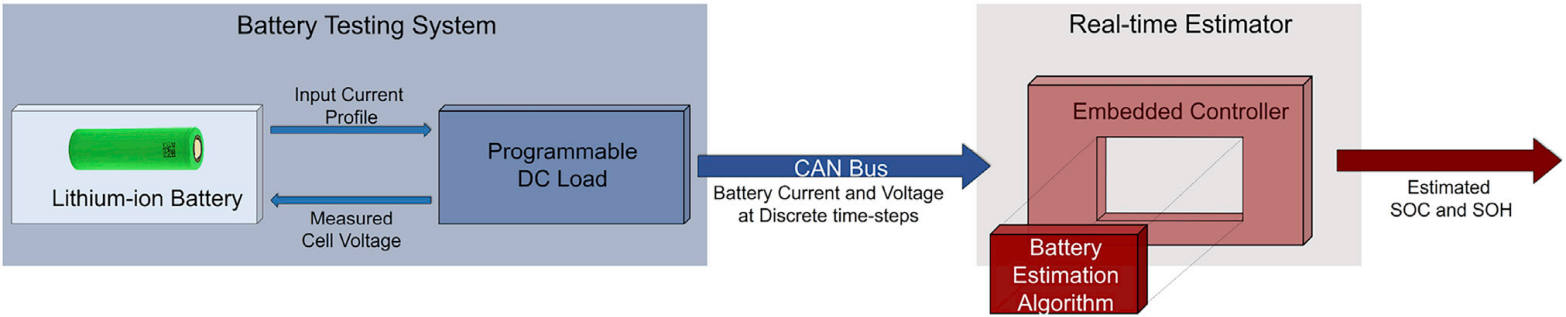
BIL Scheme General schematic of a battery-in-the-loop (BIL) setup.

In the above described HIL setup, if the hardware simulating the plant dynamics is replaced with a real physical system, which in the context of this paper is a lithium-ion battery, then it is referred to as the BIL test. It follows that the code hosted on the embedded controller is that of the estimation algorithm. BIL is a more powerful validation approach than the HIL, wherein the developed estimation algorithm can be tested in real time over the actual battery cell rather than its model. This test results in reduced development time and cost as it allows the algorithm to be tested on hardware in the early stages of development. Further, this test enables an iterative process of improving and correcting the algorithm without having to spend time waiting for the HIL test stage. The main components of a BIL, as shown in Figure 1, are as follows:

1. a lithium-ion battery,
2. a programmable direct current (DC) load to charge/discharge the battery according to a predetermined input current cycle,
3. an embedded controller that hosts the estimation algorithm, and
4. a controller area network (CAN) bus to transmit the measured current, voltage, and temperature data from the DC load to the controller. In this work, the lithium-ion battery is a cylindrical 2Ah cell with a positive electrode of nickel managanese cobalt (NMC) oxide and a negative electrode of graphite. The programmable DC Load is an Arbin LBT21024 for cycling battery systems, and the embedded controller is a dSPACE MicroAutoBox-II.

### Related Literature and Contributions

In the battery modeling and estimation literature, HIL and BIL testing has been limited to the use of battery equivalent circuit models (Song et al., 2013; Kim et al., 2014a, 2014b; Zhang et al., 2018; Tara et al., 2013) and estimation algorithms ranging from unscented Kalman filters (He et al., 2016), adaptive H-infinity filters (Zhang et al., 2016), dual H-infinity filters (Chen et al., 2017) to adaptive extended Kalman filters (Zhang et al., 2017). BIL experiments have been used, wherein a real physical battery system is connected to a controller, to evaluate energy management strategy algorithms for plug-in hybrid and hybrid electric vehicles (Kim et al., 2014a, 2014b; Zhang et al., 2018; Tara et al., 2013). Battery equivalent circuit models hosted in an HIL platform are used for testing commercial BMS controllers (Barreras et al., 2016; Dai et al., 2013). Moreover, for the validation of battery state estimation algorithms, a HIL experimental platform has been used to estimate SOC (He et al., 2010, 2016; Zhang et al., 2016, 2017; Chen et al., 2017), capacity (SOH) (Chen et al., 2017), and state of energy (Zhang et al., 2016). The embedded controllers used in the literature range from dSPACE simulators/Autobox (Song et al., 2013) (Barreras et al., 2016), real time operating system (RTOS) μCOS-II platform (He et al., 2016), and xPC Target (Zhang et al., 2016) (Zhang et al., 2017). It is to be noted that every implementation of a battery model in the HIL and BIL platforms found in the open literature is based on a battery equivalent circuit model. However, with the need for advanced BMS designs in the future, it is prudent to harness the strengths of rich physics-based modeling and control theory tools by exploring the implementation of battery electrochemical models and other types of model-based observers. To that end, the key contributions of the proposed work are as follows:

1. implementing a real-time validation approach for an electrochemical model-based adaptive interconnected observer for the combined estimation of SOC and SOH (Allam and Onori, 2020a, 2020b) showing that it is computationally feasible,
2. describing the steps to be undertaken to establish a BIL experimental setup with state-of-the-art equipment, and
3. demonstrating the real-time performance of the estimation algorithm in terms of robustness, under noisy input signals and over different drive cycles.

## Results and Discussion

### Electrochemical Model

**Figure 2:**
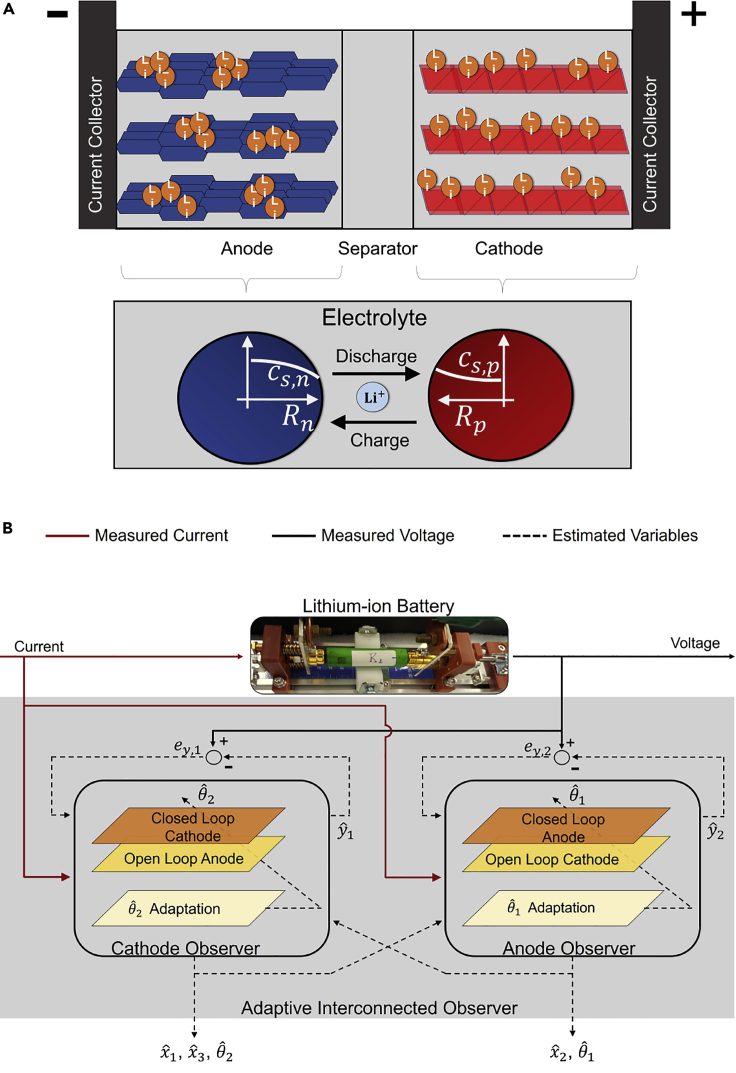
Components of an Electrochemical Model-based Adaptive Interconnected Observer (A and B) (A) Representation of a lithium-ion cell and the schematic of a single particle model (See Table S1 in the Supplemental Information for values of the SPM parameters), and (B) the interconnected adaptive observer structure for the estimation of lithium concentration states (${\hat{x}}_{1},{\hat{x}}_{2}$), total cell capacity (${\hat{x}}_{3}$), anode diffusion coefficient (${\hat{\theta }}_{1}$), and the SEI layer ionic conductivity (${\hat{\theta }}_{2}$).

The lithium-ion battery electrochemical model is characterized by a system of partial differential algebraic equations describing the transport of lithium in the solid and electrolyte phase via mass and charge conservation laws (Doyle et al., 1993). This model, popularly known as the pseudo-two-dimensional (P2D) model, is a high-dimensional and high-fidelity electrochemical model, which was traditionally used for battery design and modeling purposes. However, in this work, a low-fidelity reduced-order electrochemical model derived from the P2D model, referred to as the single particle model (SPM), is used with an aim to lend itself to observer design for online state/parameter estimation and to minimize computational effort, thereby enabling the model to be run on real-time embedded controllers that have limited power capability and resources. The SPM assumes that each electrode can be abstracted by a single spherical particle (Santhanagopalan et al., 2006), as shown in Figure 2A. Further, a uniform current density is assumed in each electrode, and dynamics in the electrolyte phase are neglected. These assumptions ensure that the SPM has a lower computational burden compared to the full-order model (P2D) at the cost of accuracy. In particular, due to neglecting the electrolyte dynamics, the model's performance at higher C-rates (wherein C-rate is defined as the rate of current in normalized form, $C-rate=I_{batt}/Q_{nom}$, where $I_{batt}$ is the applied current and $Q_{nom}$ is the nominal battery capacity) is inaccurate.

The SPM predicts the voltage behavior of the battery when the cell is at the beginning of its life. However, as the battery ages, this expression will fail to reproduce the exact degrading behavior of the cell. To that end, this paper aims to incorporate terms that explicitly depend on degradation into the SPM formulation. This work assumes that the solid electrolyte interphase (SEI) layer growth is the major degradation mechanism in lithium-ion batteries. The SEI layer growth is due to the electrolyte solvent reduction at the interface of the negative electrode and electrolyte, which consumes cyclable lithium ions, thereby reducing the cell capacity and power capability (increased resistance). The relationship between capacity fade and power fade due to SEI layer growth is exploited to derive terms that can be incorporated into the SPM. Therefore, the voltage predicted by the newly formulated aging-enhanced SPM will replicate the waning performance of the battery as it ages.

For the real-time implementation of the SPM, the PDEs describing the transport of lithium in the solid phase are spatially discretized using the finite difference method to obtain a system of ordinary differential equations (ODEs) for both electrodes. Subsequently, the ODEs and the algebraic equations are reformulated into a state-space formulation, which is expounded in the Transparent Methods in the Supplemental Information.

### Model-Based Observer

In most physical systems, not all the internal state variables can be directly measured via sensors. In such cases, model-based observers can be employed to use the system inputs and outputs, which are available via sensor measurements, to estimate the non-measurable internal states of a system. This, however, is contingent on the system's observability, wherein observability is a fundamental property of the system that guarantees that the internal states of a system can be inferred based on output sensor measurements. It follows that if a system is observable, then the internal state variables can indeed be reconstructed using the output measurements. Furthermore, state observers can be generally classified into two types: open-loop observer and closed-loop observer. The open-loop observer estimates the internal states, from a given set of initial conditions, by simulating the model without using the actual output sensor measurements. The major issue with this approach is that the error between model output and the measurements is never accounted for, and hence, any incorrect initial conditions will be propagated in time and not be corrected, resulting in state estimates that diverge from the true values. On the other hand, the closed-loop observer measures the error between the model output and sensor measurements and applies a gain proportional to this error to correct the estimated states such that the measurement error is driven to zero. This ensures that despite incorrect initialization of states, the inclusion of this feedback mechanism forces the closed-loop observer to estimate the states accurately over time.

In the context of electrochemical model-based observers for battery state estimation, the internal non-measurable states (such as lithium concentration in cathode and lithium concentration in anode) need to be inferred from the cell output voltage measurements. The weak observability of the battery system while simultaneously trying to estimate the lithium concentration in both electrodes is studied in the literature (Di Domenico et al., 2010; Bartlett et al., 2016), and the different methods to overcome the observability issues and their respective drawbacks are documented (Allam and Onori, 2018). One of the methods reports that the system is observable from the cell voltage measurements under the assumption that the lithium concentration in one electrode is simulated in open-loop fashion while the lithium concentration in the other electrode is estimated in closed-loop fashion (Bartlett et al., 2016). This, of course, assumes that the initial conditions of the open-loop model are perfectly known, which eventually allows the closed-loop estimates to converge to their true values. This approach will not work in realistic scenarios where the initial lithium concentration in both electrodes is unknown, which will result in the state estimates from the single electrode observer to diverge from the true values over time.

The idea of a single electrode observer is extended in this work by introducing dual observers, wherein a single electrode observer is dedicated for each electrode, hereby referred to as a cathode observer and an anode observer. In the cathode observer, the lithium concentration in the cathode is estimated in a closed-loop fashion while the lithium concentration in the anode is estimated in an open-loop fashion. Thus, the output error between the cathode observer's output and the cell voltage measurements is accounted for while estimating the cathode concentration states. Likewise, the structure of the anode observer is similar except that the lithium concentration in the anode is estimated in closed-loop fashion while the lithium concentration in the cathode is estimated in an open-loop fashion. Clearly, the open-loop models of both observers are still susceptible to providing erroneous values under incorrect initial conditions, which will result in faulty closed-loop estimates. This issue is overcome by realizing a bidirectional interconnection between the two observers that can correct the open-loop models over time. The closed-loop estimate of cathode's lithium concentration from the cathode observer is fed to the anode observer to correct the open-loop model of the cathode, whereas the closed-loop estimate of anode's lithium concentration from the anode observer is fed to the cathode observer to correct the open-loop model of the anode. This bidirectional interconnection ensures that despite incorrect initialization of the concentration states in both electrodes, the open-loop models in both observers are updated and corrected, which ultimately ensures that the closed-loop estimates will converge to their respective true values.

While the interconnected observer described above can concurrently estimate the lithium concentration in the cathode and the anode (Allam and Onori, 2018) to provide information on SOC, it is not equipped to estimate SOH indicators like the cell capacity (SOH). This is because as battery ages, some of the electrochemical model parameters identified at the beginning of life will change due to battery degradation mechanisms. Using such a model for estimation purposes throughout the entire lifespan of the battery will result in state estimates (SOC and SOH) to diverge since the model will no longer remain accurate with aging. Hence, an adaptation mechanism is considered to ensure the aging-sensitive model parameters are adapted as the battery degrades, resulting in an adaptive interconnected observer. This ensures that the combined estimation of the states and parameters remain accurate despite aging.

To that end, an adaptive interconnected observer (Allam and Onori, 2020a, 2020b), as shown in Figure 2B, is developed to concurrently estimate the lithium concentration in both electrodes, cell capacity, and aging-sensitive model parameters, despite any incorrect initialization of states and parameters. An aging-enhanced SPM, introduced earlier, is used as a basis to develop the observer, and the choice of observer structure is a sliding mode observer, which is a class of robust observers that can handle model uncertainties via variable structure gains. The sensor measurements of current and voltage of a lithium-ion cell act as an input to the adaptive interconnected observer. The cathode observer estimates the lithium concentration in the cathode $\left({\hat{x}}_{1}\right)$, cell capacity $\left({\hat{x}}_{3}\right)$, and the SEI layer ionic conductivity $\left({\hat{\theta }}_{2}\right)$. The anode observer estimates the lithium concentration in the anode $\left({\hat{x}}_{2}\right)$ and the anode diffusion coefficient $\left({\hat{\theta }}_{1}\right)$. Further, the practical stability of the adaptive interconnected observer's estimation error dynamics has been proved analytically (Allam and Onori, 2020b), which ensures that the estimated variables converge around the respective true values within a bounded error ball of radius defined by the uncertainties in the aging-enhanced SPM.

It has to be pointed out that the continuous-time adaptive interconnected observer formulation (Allam and Onori, 2020b) cannot be directly implemented on an embedded controller. Since the sensor measurements in a real system are available at discrete sample times, the continuous-time system needs to be sampled at particular time intervals to obtain a discrete-time system. The discrete-time formulation of the adaptive interconnected observer (reported in the Transparent Methods in the Supplemental Information) can be readily implemented on an embedded controller in real time for BIL experiments.

### BIL Experimental Results

Experiments are conducted on a cylindrical lithium-ion cell with graphite at the negative electrode and NMC at the positive electrode. The cell capacity measured using the manufacturer's recommended discharge current of 1C is 1.95Ah. The cell is introduced into the BIL setup as shown and described in the Transparent Methods in the Supplemental Information (See Figures S1 and S2 for experimental setup and connections, Tables S2 and S3 for specification of the cell and equipment). The cell is subjected to dynamic current profiles, such as the Urban Dynamometer Driving Schedule (UDDS) and the world harmonized light-duty vehicles test procedure (WLTP), through MITS PRO software and the Arbin LBT21024 equipment. The Arbin system measures the current and voltage of the battery and transmits them via Arbin's CAN port every 0.1s, which is received by the CAN port of the dSPACE MicroAutoBox-II (see Figures S3–S5, and Table S4 in Transparent Methods in the Supplemental Information for relevant CAN communication specifications). These received signals are fed as an input to the adaptive interconnected observer running in real time hosted by the MicroAutoBox.

To demonstrate the robustness of the observer, the model is incorrectly initialized to verify if it can converge to the true values despite the incorrect initialization and in the presence of sensor noises and corrupted input signals. The estimated variables by the observer, which are the SOC and cell capacity (SOH), are compared with the true SOC value from the battery computed using the coulomb counting method and to the true measured capacity of the battery (1.95Ah), respectively. In addition, the estimated variables from these BIL results are also compared to the MIL test results which are performed via offline simulation with no computational constraints or input signal corruption. This comparison with measured true benchmark values allows the user to evaluate the performance of the real-time effectiveness of an observer.

**Figure 3:**
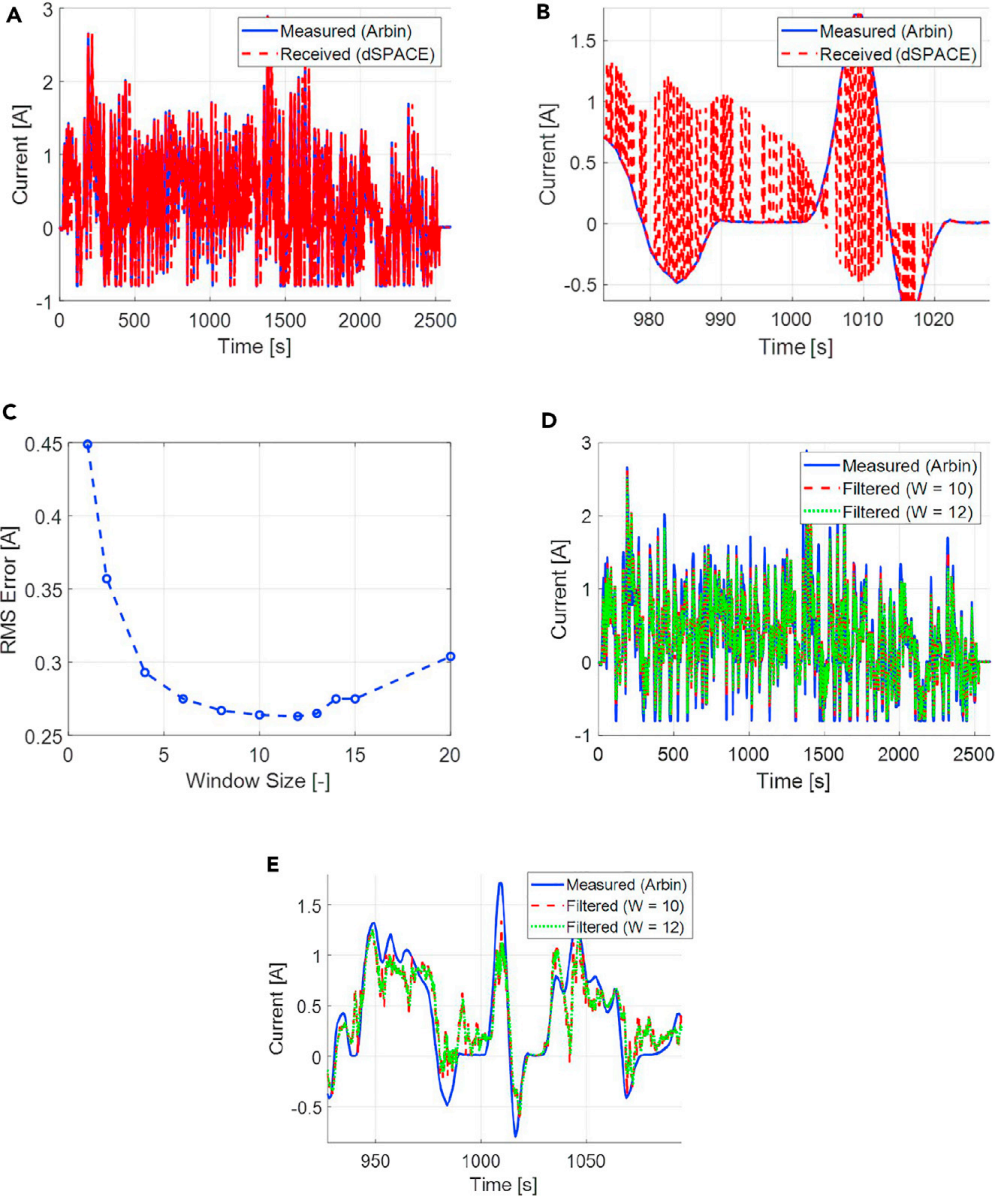
Observer Input Signal Corruption and Correction (A–E) (A) Signal comparison between measured current by the Arbin and the corrupted signal received at the dSPACE MicroAutoBox via CAN bus, (B) zoomed-in version of the current comparison plot, (C) the RMSE plot for different window sizes for the moving average filter, (D) the comparison of measured current by the Arbin and the filtered signal at the dSPACE MicroAutoBox, and (E) the zoomed-in version of the measured current and filtered current plot.

It is noted that the signals (current, voltage) transmitted over the CAN bus from the Arbin to the dSPACE MicroAutoBox every 0.1s are corrupted with quantization errors. It is understood that due to the fast sampling time of 0.1s, the signal received at the MicroAutoBox is highly corrupted, as shown in Figures 3A and 3B. As a result, a moving average filter is introduced in the MicroAutoBox to smooth out the signals before feeding them as an input to the adaptive observer. The window size of the moving average filter is chosen to be 10. This size is chosen after running the experiments for various window sizes and selecting the one which outputs a filtered signal that is closest to the measured signals (the closeness to the measured signal is verified by computing the RMS error), as shown in Figure 3C. The resulting filtered signal is well constructed and an approximate version of the actual measured current signal, as shown in Figures 3D and 3E, which is then fed to the observer algorithm hosted in the MicroAutoBox.

**Figure 4:**
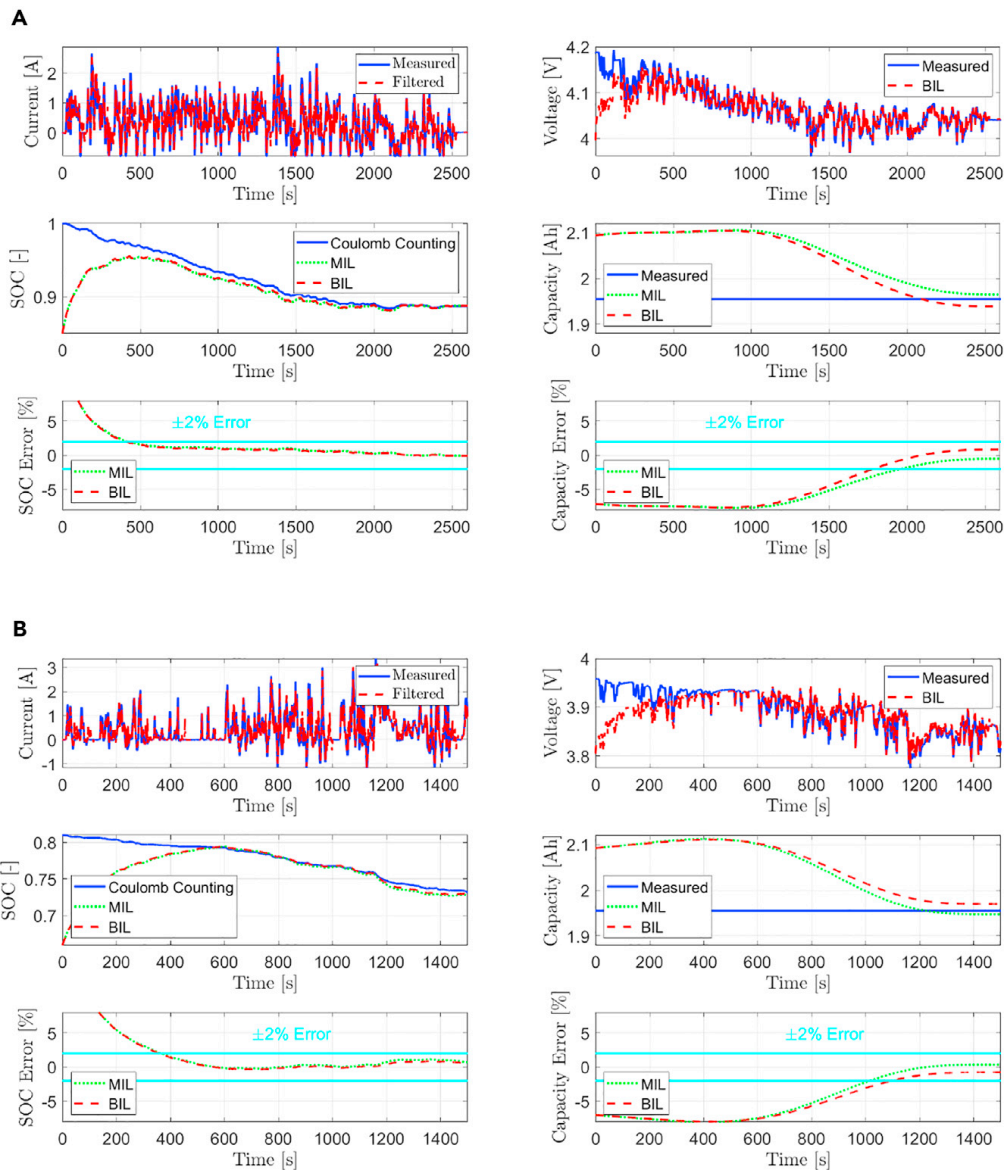
Battery SOC/SOH Estimation Results (A and B) SOC/SOH estimation results for the (A) UDDS and (B) WLTP input current profiles. The plot shows the input current signal, voltage, SOC and capacity estimation results, and the estimation error plots for SOC and capacity.

The results for the UDDS and the WLTP profiles are shown in Figures 4A and 4B, respectively. For both cases, the lithium concentration (SOC) states are initialized with an error of 15%, and the capacity (SOH) state is initialized with an error of 7%. The cell is fully charged before subjecting it to the UDDS profile, whereas the cell is at 80% SOC before applying the WLTP profile. In Figures 4A and 4B, the legend MIL refers to the estimated states by the adaptive observer in an offline simulation environment, where the input to the observer is fed from the measured experimental data and not the filtered input signals, whereas BIL refers to the estimates from the real-time validation with the physical battery. Note that the initial SOC estimation error is higher during the initial phase of the experiments because the observer is incorrectly initialized. The cathode concentration state variables (which makes up the bulk SOC) are initialized with an error of 15% at the beginning to verify if the observer can overcome this initial incorrect error and still converge with the true value over time. As observed, the BIL estimation is comparable to the MIL despite corrupted input signals. The results further validate the practical stability notion by showing that the SOC and capacity estimates always stay bounded within the ±2% error with respect to the reference/measured values.

### Conclusions

In this work, the importance of real-time validation approaches for battery estimation algorithms is motivated and a description of the BIL experimental setup is presented. The paper focuses on pushing the envelope in battery estimation algorithms by implementing an electrochemical model-based observer in real time, thereby bridging the gap between theory and real-time hardware validation without compromising on the performance. Every component required to setup the BIL experiments is described in detail, and the steps to establish the communication between a physical lithium-ion cell and the embedded controller that hosts the adaptive observer are outlined. The BIL validation of the adaptive observer is performed over two dynamic driving profiles – UDDS and WLTP. The SOC and SOH estimation results from the BIL are always bounded within ±2% of their respective true values, despite the corruption of the input signal in real time. The validation demonstrates that an advanced electrochemical model-based estimation algorithm can be run in real time with good accuracy against noises and errors induced due to real physical connections. The use of such algorithms in real-time applications holds the key to improve battery lifespan, enable accurate diagnosis/prognosis, and identify cells of similar health for the feasibility of using them in “second-use” applications upon retirement.

### Limitations of the Study

The proposed adaptive interconnected observer estimates the cell capacity (SOH) by exploiting the relationship between power fade and capacity fade due to the growth of the SEI layer at the anode. This observer assumes that SEI layer growth is the primary degradation mechanism plaguing lithium-ion batteries. While this may be true for certain applications, as cells continue to age and based on their usage, they may undergo different degradation mechanisms such as lithium plating. It is worth understanding how effects of other degradation mechanisms can be incorporated into the aging-enhanced SPM to more accurately estimate the cell capacity in the presence of degradation caused due to mechanisms other than SEI layer growth.

Further, the work presented in this paper is for the SOC/SOH estimation of individual cells. Future work will involve extending it to a battery pack that has been aged over multiple cycles, which is composed of multiple lithium-ion cells connected in series and/or parallel, to verify the effectiveness of the observer for large-scale battery packs in real time.

### Resource Availability

#### Lead Contact

Further information and requests should be directed to and will be fulfilled by the Lead Contact, Simona Onori (sonori@stanford.edu).

#### Materials Availability

This study did not generate new materials.

#### Data and Code Availability

The data and code supporting the current study is available from the Lead Contact on request.

## Methods

All methods can be found in the accompanying Transparent Methods supplemental file.

## Supplemental Information

Document S1. Transparent Methods, Figures S1–S5, and Tables S1–S4

## Footnotes

Supplemental Information can be found online at https://doi.org/10.1016/j.isci.2020.101847.
