# Supplementary material for: Pushing the Eenvelope in Battery Estimation Algorithms
Source: iScience. 2020 Nov 23;23(12):101847. doi: 10.1016/j.isci.2020.101847 (PMC7721640; doi:10.1016/j.isci.2020.101847)
Supplement: Document S1. Transparent Methods, Figures S1–S5, and Tables S1–S4 [file mmc1.pdf]

iScience, Volume *23*

Supplemental Information

Pushing the Eenvelope in Battery Estimation Algorithms

Anirudh Allam, Edoardo Catenaro, and Simona Onori

Supplemental Information

Transparent Methods

Experimental Procedure

Experiments were carried out at the Stanford Energy Control Laboratory, in the Energy Resources Engineering Department, Stanford University. The equipment components, labeled in Fig. S1A, include an host computer used to program test profiles and real-time data monitoring through the MITS Pro and Data Watcher software (label 1), the Arbin LBT21024 with a programmable power supply (label 2), a battery cell positioned in a high-current cylindrical cell holder (maximum current of 200A) manufactured by Arbin (label 3), the Arbin measurement system (label 4), an embedded controller dSPACE MicroAutoBox-II (label 5), and a dSPACE Control Desk software to supervise dSPACE simulator real-time data (label 6), which provides a platform to test in real-time the design of control/estimation algorithms. The BIL architecture relies on a CAN BUS connection between battery (Arbin system) and dSPACE controller (ECU), represented in Fig. S1A.

Data communication between different equipment components is outlined in Fig. S1B and described as follows. The input current profile is configured via the MITS Pro software, which is transmitted through the TCP/IP connection to the Arbin LBT21024, before being subjected to the cell under test. The behavior of the cell in response to the current stimuli is measured by the Arbin LBT21024 in the form of voltage response. The measured current and voltage signals are sent by Arbin LBT21024 to the Arbin measurement system through the TCP/IP connection, which are then transmitetd through the CAN bus to the dSPACE MicroAutoBox-II controller. In order to access and monitor dSPACE MicroAutoBox-II real-time data for processing, data are transmitted through TCP/IP to the host computer equipped with dSPACE ControlDesk software.

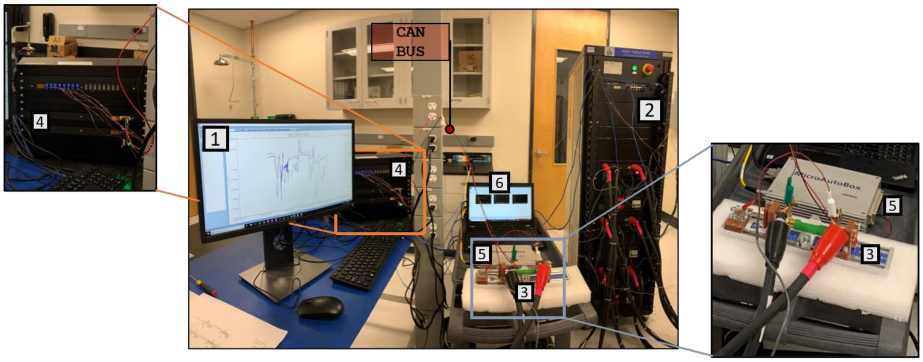

(A)

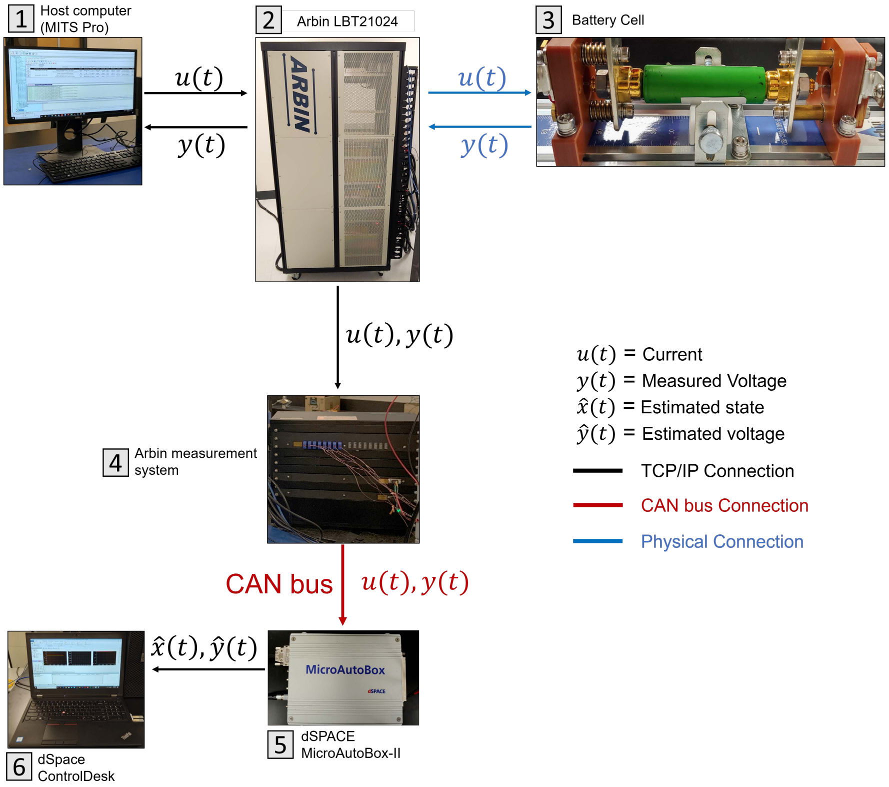

(B)

Figure S1: (A) Experimental setup at the Stanford Energy Control Laboratory. (B) Communication signals between each laboratory equipment component. Related to the physical/experimental realization of Figure 1.

Electrochemical Modeling

The conservation of mass in the solid phase governs the transport of lithium in the solid phase of each electrode ($j = [n, p]$, where n stands for negative electrode and p is for positive electrode) described by the following PDE and its respective boundary conditions

$$\begin{cases} \dfrac{\partial c_{s,j}}{\partial t} = D_{s,j}\left[\dfrac{2}{r}\dfrac{\partial c_{s,j}}{\partial r} + \dfrac{\partial^2 c_{s,j}}{\partial r^2}\right] \\ \dfrac{\partial c_{s,j}}{\partial r}\Big|_{r=0} = 0; \quad \dfrac{\partial c_{s,j}}{\partial r}\Big|_{r=R_j} = \dfrac{\pm I_{batt}}{F a_{s,j} D_{s,j} A L_j}. \end{cases} \tag{1}$$

The overpotential of each electrode is obtained from the Butler-Volmer kinetic equation that describes the rate of intercalation and de-intercalation of lithium ions as

$$\eta_j = \frac{R_g T}{0.5F} \cdot \sinh^{-1}\left(\frac{I_{batt}}{2a_{s,j} A L_j i_{0,j}}\right) \tag{2}$$

where the exchange current density $i_{0,j} = F k_j \sqrt{c_{e,0} c_{s,j,surf}\left(c_{s,j,max} - c_{s,j,surf}\right)}$. By exploiting the relationship between capacity and power fade due to SEI layer growth at the anode, an aging-enhanced expression for the cell terminal voltage is derived as (Allam and Onori, 2020)

$$\begin{aligned} V = & \left[U_p\left(c_{s,p,surf}\right) + \eta_p\left(c_{s,p,surf}, I_{batt}\right)\right] - \\ & \left[U_n\left(c_{s,n,surf},\right) + \eta_n\left(c_{s,n,surf}, I_{batt}\right)\right] - \\ & - I_{batt}(t) R_{e,0} - I_{batt}(t) R_l - I_{batt}(t) R_{pf}(t), \end{aligned} \tag{3}$$

where U_j is the open circuit potential of the electrode which is a function of the stoichiometry ratio, θ_j that depends on the respective surface concentration as $\theta_j = c_{s,j,surf}/c_{s,j,max}$, and $R_{e,0}$ is the initial electrolyte resistance at the beginning of life expressed as (Di Domenico et al., 2010)

$$R_e = \frac{1}{2A}\left(\frac{L_n}{\kappa_n \epsilon_{e,n}^{1.5}} + \frac{2L_s}{\kappa_s \epsilon_{e,s}^{1.5}} + \frac{L_p}{\kappa_p \epsilon_{e,p}^{1.5}}\right). \tag{4}$$

For the real-time implementation of the aging-enhanced SPM, the Partial Differential Equation (PDE) describing the transport of lithium in the solid phase,

given in (1), is spatially discretized using the Finite Difference Method (FDM) into $N+1$ concentration nodes. The resulting system of Ordinary Differential Equations (ODEs) for both electrodes of the dimension $2N$ is represented using a state-space model for the ease of observer development and implementation. The state vector is considered to be $x = [x_1, x_2, x_3,]^T \in \mathbb{R}^{(2N+1)\times 1}$, $u = I_{batt}$ is the input current, and $y = V$ is the cell terminal voltage. The state variables represent lithium concentration in cathode $x_1 = [c_{s,p,1}, c_{s,p,2}, \ldots, c_{s,p,N}]^T$, anode $x_2 = [c_{s,n,1}, c_{s,n,2}, \ldots, c_{s,n,N}]^T$, and cell capacity $x_3 = Q$. It is to be noted that cell capacity is not an actual state, but augmented to the state vector to enable its estimation. The dynamics of capacity degradation are slowly varying, hence the time derivative is considered zero for practical purposes $\dot{Q} = 0$. Moreover, the surface concentration in both electrodes is given as $c_{s,j,surf} = Cc_{s,j}$, respectively, where C is the output distribution vector given as $C = [0\,0\ldots 1]$. Then the state space formulation of SPM is given by

$$\begin{aligned}
\dot{x}_1(t) &= A_{11}x_1(t) + B_1 u(t) \\
\dot{x}_2(t) &= \theta_1 \bar{A}_{22} x_2(t) + B_2 u(t) \\
\dot{x}_3(t) &= 0 \\
y(t) &= h_1(x_{1,N}, u) - h_2(x_{2,N}, u) - h_3(x_3)u - \\
&\quad - R_l u + (x_3 - Q_0)\,\theta_2 u,
\end{aligned} \tag{5}$$

where nonlinearities in the terminal voltage equation, and parameters are

$$\begin{aligned}
h_1(x_{1,N}, u) &= [U_p(c_{s,p,surf}) + \eta_p(c_{s,p,surf}, I_{batt})], \\
h_2(x_{2,N}, u) &= [U_n(c_{s,n,surf}) + \eta_n(c_{s,n,surf}, I_{batt})], \\
h_3(x_3) &= R_e, \\
\theta_1 &= D_{s,n}, \\
\theta_2 &= \frac{3600 M_{sei}}{2FA^2 \rho_{sei} a_{s,n}^2 L_n^2 \kappa_{sei}},
\end{aligned}$$

and square matrices $A_{11}, \bar{A}_{22} \in \mathbb{R}^{N\times N}$ are the coefficients of the concentration states in (5), and column vectors $B_1, B_2 \in \mathbb{R}^{N\times 1}$ are coefficients of input current

in (5), as given below

$$A_{11} = \frac{D_{s,p}}{\Delta_r^2} \begin{bmatrix} -2 & 2 & 0 & \cdots & 0 & 0 \\ 1/2 & -2 & 3/2 & \cdots & 0 & 0 \\ \vdots & \vdots & \vdots & \ddots & \vdots & \vdots \\ 0 & 0 & 0 & \cdots & 2 & -2 \end{bmatrix} \tag{6}$$

$$B_1 = \frac{-2}{\Delta_r F a_{s,p} A L_p} \begin{bmatrix} 0 \\ 0 \\ \vdots \\ \frac{N+1}{N} \end{bmatrix}$$

$$\bar{A}_{22} = \frac{1}{\Delta_r^2} \begin{bmatrix} -2 & 2 & 0 & \cdots & 0 & 0 \\ 1/2 & -2 & 3/2 & \cdots & 0 & 0 \\ \vdots & \vdots & \vdots & \ddots & \vdots & \vdots \\ 0 & 0 & 0 & \cdots & 2 & -2 \end{bmatrix}$$

$$B_2 = \frac{2}{\Delta_r F a_{s,n} A L_n} \begin{bmatrix} 0 \\ 0 \\ \vdots \\ \frac{N+1}{N} \end{bmatrix}.$$

The procedure used to identify the model parameters, and validate it against experimental data is outlined (Allam and Onori, 2020). Further, the identified parameter values are listed in Table S1, and the rest of the geometrical and aging parameters are borrowed. (Allam and Onori, 2018; Prada et al., 2013). Further, note that the bulk SOC of the cell is limited by the cathode. Hence, the bulk SOC of the cell is computed by volume-averaging the concentration values at all discretization grid points in the cathode and normalizing it with respect to the cathode's maximum and minimum stoichiometry values, as given

Table S1: SPM Parameters. Related to Figure 2A.

Parameter	Value
L_n [m]	60.6×10^{-6}
L_p [m]	52.5×10^{-6}
A [m^2]	0.093
$c_{s,n,max}$ [mol m^{-3}]	27920
$c_{s,p,max}$ [mol m^{-3}]	45711
$\epsilon_n[-]$	0.53
$\epsilon_p[-]$	0.54
$D_{s,n,ref}$ [m^2 s^{-1}]	1.74×10^{-14}
$D_{s,p,ref}$ [m^2 s^{-1}]	2.98×10^{-14}
k_n[m$^{2.5}$ mol$^{-0.5}$ s^{-1}]	3.16×10^{-10}
k_p [m$^{2.5}$ mol$^{-0.5}$ s^{-1}]	5.96×10^{-10}
R_l [Ω]	0.026
L_s [m]	21×10^{-6}
$\epsilon_s[-]$	0.58

below:

$$c_{s,p,bulk} = \frac{1}{\frac{4}{3}\pi N^3} \sum_{i=1}^{N} 4\pi i^2 c_{s,p,i}, \tag{7}$$

$$SOC = \frac{\theta_{p,0\%} - \frac{c_{s,p,bulk}}{c_{s,p,max}}}{\theta_{p,0\%} - \theta_{p,100\%}} \tag{8}$$

For the numerical implementation of continuous-time systems in physical embedded controllers such as the dSPACE MicroAutoBox-II, the continuous-time systems are approximated in a discretized time fashion by selecting a sampling time or time step (Δk). The discrete-time representation of the battery model is given below:

$$x_1[k+1] = (I + A_{11}\Delta k)\, x_1[k] + B_1 u[k]\, \Delta k$$

$$x_2[k+1] = \left(I + \theta_1 \bar{A}_{22}\Delta k\right) x_2[k] + B_2 u[k]\, \Delta k$$

$$x_3[k+1] = x_3[k] \tag{9}$$

$$y[k] = h_1(x_{1,N}[k], u) - h_2(x_{2,N}[k], u) - h_3(x_3[k])u[k] - R_l u[k] + (x_3[k] - Q_0)\, \theta_2 u[k], \tag{10}$$

where $I \in \mathbb{R}^{N \times N}$ is the identity matrix, and k is the discrete sample time.

Observer Design

The adaptive interconnected sliding mode observer for combined estimation of lithium concentration (SOC), capacity (SOH), and aging-sensitive parameters consists of two parts: *cathode observer* and an *anode observer*. The two aging-sensitive parameters that are adaptively estimated are the anode diffusion coefficient (θ_1) and SEI layer ionic conductivity (θ_2), which are moderately sensitive to the measured cell voltage (Ramadass et al., 2003; Edouard et al., 2016). The parameter θ_1 appears in the anode concentration dynamics and hence it is estimated via the anode observer. On the other hand, the parameter θ_2 appears in the system output equation and hence can be estimated via either observer, anode or cathode, as per the designer's choice. In this work, the cathode observer is used to estimate the parameter θ_2, which also aids in systematically deriving the stability proof of the adaptive interconnected observer as documented in (Allam and Onori, 2020). The discrete-time formulation of a *cathode observer* is

$$
\begin{aligned}
\hat{x}_1[k+1] =& (I + A_{11}\Delta k)\hat{x}_1[k] + \big(B_1 u[k] + \\
& G_1\left(y[k] - \hat{y}_1[k]\right) + G_{v1}\,\mathrm{sgn}\left(y[k] - \hat{y}_1[k]\right)\big)\Delta k \\
\hat{x}_{2,ol}[k+1] =& \left(I + \hat{\theta}_1[k]\bar{A}_{22}\Delta k\right)\hat{x}_2[k] + B_2 u[k]\Delta k \\
\hat{x}_3[k+1] =& x_3[k] + G_3\left(y[k] - \hat{y}_1[k]\right)u[k]\Delta k \\
\hat{y}_1[k] =& h_1\left(\hat{x}_{1,N}[k], u[k]\right) - h_2\left(\hat{x}_{2,N,ol}[k], u[k]\right) - \\
& R_l u[k] - h_3(\hat{x}_3[k])u[k] + \\
& \left(\hat{x}_3[k] - Q_0\right)\hat{\theta}_2[k]u[k],
\end{aligned} \tag{11}
$$

and *anode observer* is

$$
\begin{aligned}
\hat{x}_{1,ol}[k+1] =& \left(I + A_{11}\Delta k\right)\hat{x}_1[k] + B_1 u[k]\Delta k \\
\hat{x}_2[k+1] =& \left(I + \hat{\theta}_1[k]\bar{A}_{22}\Delta k\right)\hat{x}_2[k] + \big(B_2 u[k] + \\
& G_2\left(y[k] - \hat{y}_2[k]\right) + G_{v2}\,\mathrm{sgn}\left(y[k] - \hat{y}_2[k]\right)\big)\Delta k \\
\hat{y}_2[k] =& h_1\left(\hat{x}_{1,N,ol}[k], u[k]\right) - h_2\left(\hat{x}_{2,N}[k], u[k]\right) - \\
& R_l u[k] - h_3(\hat{x}_3[k])u[k] + \\
& \left(\hat{x}_3[k] - Q_0\right)\hat{\theta}_2[k]u[k].
\end{aligned} \tag{12}
$$

In (11) and (12), the subscript *ol* stands for open loop model state variables, $G_1 \in \mathbb{R}_-^{N\times 1}, G_2 \in \mathbb{R}_+^{N\times 1}$, $G_3 \in \mathbb{R}$ are constant linear observer gains, $G_{v1}, G_{v2} \in \mathbb{R}^{N\times 1}$ are variable structure gains, introduced to improve robustness against uncertainties, with discontinuous injection terms defined as

$$
\mathrm{sgn}\left(y - \hat{y}_i\right) = \begin{cases} 1, & \text{if } y - \hat{y}_i > 0 \\ 0, & \text{if } y - \hat{y}_i = 0 \\ -1, & \text{if } y - \hat{y}_i < 0. \end{cases} \quad i = 1, 2.
$$

The estimation error dynamics of the aforementioned adaptive interconnected sliding mode based observer is proved using Lyapunov's stability theory.(Allam and Onori, 2020)

BIL Experimental Setup

For the BIL experiments, a cylindrical lithium-ion cell is investigated, whose representation and manufacturer specifications are reported in Table S2. Further, the technical specifications of the BIL components introduced in Section are provided in Table S3.

Chemistry Composition	$LiNiMgCoO_2$/graphite
Manufacturer	Sony
Manufacturer Model	US18650VTC4
Diameter x Length [mm x mm]	18.35 x 65.2
Weight $M_{nom,cell}$ [g]	45
Nominal Voltage V_{nom} [V]	3.7
Nominal Capacity Q_{nom} [Ah]	2.1
Charging Voltage V_{charge} [V]	4.2
Charging Current I_{charge} [A]	2
Cut-off Voltage V_{cutoff} [V]	2.5
Maximum Voltage V_{max} [V]	4.2

Table S2: Manufacturer specifications for the cylindrical, fresh NMC cell used in this work. Related to Figure 2A.

The key element is establishing the BIL is the CAN bus communication between the battery testing system and the embedded controller, which is implemented both in terms of hardware and software. The CAN is a serial communication protocol used in automobiles for efficient and high speed transfer of electrical signals between multiple ECUs. The information exchange between ECUs is in the form of messages with a unique identifier (or ID) that contain the values of physical variables. In this paper, CAN messages containing the cell current and voltage information are transmitted from the Arbin battery measurement system (CAN channel 1) to the dSPACE AutoMicroBox-II (CAN

Laboratory Equipment	Manufacturer Technical Specifications	
Arbin battery test system	Manufacturer	Arbin Instruments
	Model	LBT21024
	Number of Channels	6
	Voltage Range [V]	$0-5$
	Current Ranges [A]	±0.5, ±5, ±50 and ±250
	Maximum Continuous Output Power [W]	1250
	Measurement Resolution	24-bit
	Simulation Control	Current/Power Simulation
	Auxiliaries	Temperature Measurement
	AC Power Input	3-Phase 50/60Hz 208VAC
		Input Power: 17400VA
Arbin Measurement System	Manufacturer	Amerex Instrument
	Model	LBT21024
	Voltage [V]	$90-264$
	Max. Current	220V 1.6A / 110V 3.2A
	Max Power [VA]	350
	Phase	1
dSPACE MicroAutoBox-II 1401/1513	Manufacturer	dSPACE
	Processor	IBM PPC 750GL, 900 MHz
		(incl. 1 MB level 2 cache)
	Main Memory [MB]	16
	Boot Time	1MB application, 160 ms
		3MB application, 340 ms
	CAN Interface	6 CAN channels
	Input/output Resolution	32 16-bit ch./8 16-bit ch.
	Input and output voltage range [V]	-10,+10

Table S3: Technical specifications of the Arbin battery test system, Arbin measurement system, dSPACE Scalexio simulator and dSPACE MicroAutoBox-II 1401/1513. Related to Figure 1.

channel 1).

The CAN hardware implementation via physical wiring is visualizd in Fig. S2. On the Arbin battery measurement system side, a D-Sub connector (model 171-009-113R911) is used to setup the electrical connection. The low and high voltage CAN pins of the D-Sub connector, CAN_L and CAN_H, respectively, are connected to the high and low voltage CAN pins of the dSPACE AutoMicroBox-II CAN channel 1.

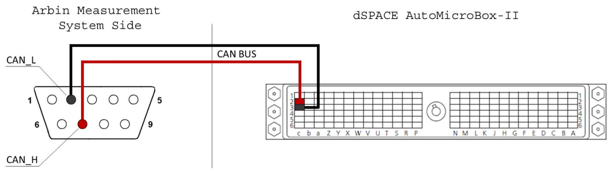

Figure S2: CAN bus connection between the Arbin battery measurement system and the dSPACE AutoMicroBox-II. The red wire (high voltage) is connected to the CAN_H pin of the D-Sub connector (Arbin battery measurement system side) and to the CAN 1 high on the dSPACE AutoMicroBox-II side. In the same way, the black wire (low voltage) is connected to the CAN_L pin and to the CAN 1 low. Related to Figure 1.

The CAN software implementation involves configuring the CAN messages, setting the baud rate, and finalizing the transmission and receiving frequency of the messages. The necessary steps are outlined below:

1. **CAN bus configuration with MITS Pro software:** This step addresses the activation of the CAN communication channel on the Arbin battery system. The MITS Pro Software, installed on the host computer labeled by 1 in Fig. S1A, is used to adjust communication settings. The current profile to be applied to the battery cell is configured in the *Schedule Files* window of MITS Pro. The current profile is composed of a rest period, wherein the cell is soaked in the desired ambient temperature of $23°C$ during a one hour rest time period (input current set to 0), and a dynamic current profile (UDDS or WLPT drive cycle). The CAN communication settings are established by opening the *CANConfig Files* window, as shown in Fig. S3A, and setting the message properties and baud rate. A new CAN configuration file, called CANconfig_Arbin_Dspace.can, is created and the sub-windows of which are described as follows:
   - *CAN Global*: the baud rate is set to 500K.
   - *Inbound CAN Signal Configuration*: Meta-variables CAN_MV_RX1 and CAN_MV_RX2, voltage and current signals, respectively, are

considered. They are transmitted through CAN message data, whose structure is defined in Fig. S3A. Note that the CAN message structure must be consistent between the transmitter and receiver system (in this paper, they correspond to the Arbin battery system and the MicroAutoBox, respectively). The *CAN Message ID* is defined as 0x100 and the *Byte Order* is set to *Little Endian.* The current and voltage signals are each 4 bytes, and hence the *Start Byte Index*, *End Byte Index*, *Start Bit Index* and *End Bit Index* are set to 0, 0, 3, 7 for the voltage message and 4, 0, 7, 7 for the current message.

- *IV Outbound CAN Message Broadcasting*: The broadcasting CAN message ID is again set to 0x100, and the desired *CAN Message Interval* is set to 100 [ms] in Fig. S3B.

Lastly, the finalized CAN configuration file CANconfig_Arbin_Dspace.can is assigned to the Arbin channel that is connected to the cell under test in the *Batch Files* window of the MITS Pro. As shown in Fig. S4, the cell under test is connected to Arbin channel 1 (*Channel index* 1), the corresponding UDDS current *Schedule* file is assigned (US18650VTC4_T23_Arbin_-Dspace_demo_UDDS.sdu), the CANconfig_Arbin_Dspace.can file is assigned to the *BMS CAN Signal Configuration File*, and the *Battery Name* is set to US18650VTC4 (which refers to battery specifications related to the ell under test reported in Table S2). The *Launch Monitor Control* window in MITS Pro launches the experiment.

2. **Real-time Interface (RTI):** The dSPACE-Simulink RTI library allows the interface between the discrete-time model-based observer implementation on Simulink with the physical input/output hardware ports of the dSPACE MicroAutoBox-II. In order to properly receive and read the CAN messages at the dSPACE AutoMicroBox-II end, which are transmitted by the Arbin system, the RTI blockset is utilized. The proposed Simulink block scheme, shown in Fig. S5, enables the embedded controller to re-

	Meta Variable Name	Nick Name	Enable	Data Log	CAN Message ID	DLC of CAN Message	Byte Order	DataType	Start Byte Index	Start Bit Index	End Byte Index	End Bit Index	Value Offset	Value Scale Factor	Unit
1	CAN_MV_RX1	Voltage	☑	Interval 500ms	0x100	8	Little Endian	Float	0	0	3	7	0	1	V
2	CAN_MV_RX2	Current	☑	Interval 500ms	0x100	8	Little Endian	Float	4	0	7	7	0	1	A

(A)

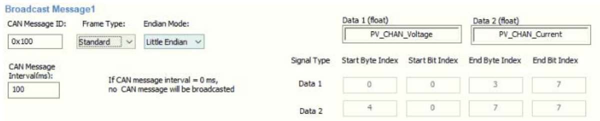

(B)

Figure S3: Tabs included in the MITS Pro software *CANConfig Files* window: (A) *Inbound CAN Signal Configuration*, describing how CAN messages are structured, and (B) *IV Outbound CAN Message Broadcasting*, which determines the broadcasting CAN message interval. Related to Figure 1.

Channel Index	Schedule	BMS CAN Signal Configuration File	Battery Name	Battery Type ID
1	US18650VTC4\US18650VTC4_T23_Arbin_Dspace_demo_UDDS.sdu	CANConfig_Arbin_Dspace.can	US18650VTC4	1

Figure S4: MITS Pro software *Batch Files* window used to assign the Arbin channel to the scheduled profile, CAN signal configuration file and battery type. Related to Figure 1.

ceive the CAN messages from the Arbin battery system that contain the real-time battery current and voltage measurements. The RTI blocks in Fig. S5 are explained below

- The *RTI Data* block is a standard block used to define the dSPACE RTI environment in Simulink.
- The *CAN Controller Setup* block is used to define the CAN controller specifications. The block settings that need to be adjusted are in the *Unit* tab: the *Mudule* is set to *CAN Type 1*, the *Controller number* to 2, the *GroupId* to *RTICAN2* and the Baudrate to 500 [kbit/s].
- the *CAN Receive Message* block determines the CAN message structure which needs to match the one previously defined for the Arbin battery system in the MITS Pro software. In the *Message* tab, the *Message identifier* is set to standard (STD), hexadecimal (hex) and 0x100, and the *Message length* is set to 8 bytes. The second tab called

Message Composition describes the process by which the model can recognize the current and voltage data within the CAN message. The composition propoerties for both signals are shown in Table. S4. In summary, using the RTI library, the properties of the CAN message to be received are set by ssigning the CAN message length to 8 bytes, corresponding to 64 bits, which is split in two parts: the first containing cell's voltage signal and the second part carrying the cell's current signal information.

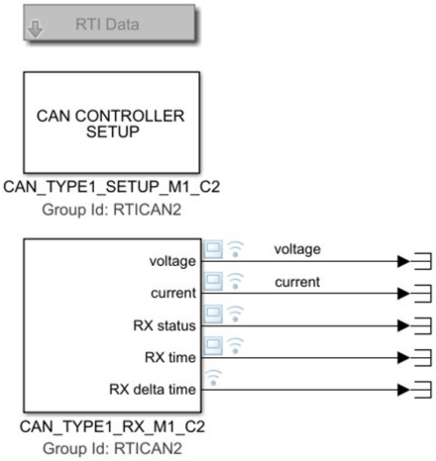

Figure S5: RTI Simulink block scheme. Related to Figure 1.

Upon configuring the RTI CAN blocks and interfacing it with the estimation algorithms, the code of the Simulink model is built and downloaded into the target platform, which is the dSPACE AutoMicroBox-II. As soon as the code is successfully flashed, the dSPACE AutoMicroBox-II light turns green. Then Arbin system measurements (current and voltage) are transmitted in real-time to the dSPACE AutoMicroBox-II through the CAN bus. Furthermore, a system description file (*.sdf), which is used in the ControlDesk software to control and visualize variables in real-time (further described in the next step).

	Current signal	Voltage signal
Signal name	current	voltage
Start bit	0	32
Signal length	32	32
Signal type	Standard	Standard
Data type	float32(IEEE)	float32(IEEE)
Byte layout	Little endian	Little endian
Factor	1	1
Factor	0	0
Physical unit	A	V

Table S4: Voltage and current signal message composition. Related to Figure 1.

3. **Real-time data monitoring on dSPACE ControlDesk:** The dSPACE ControlDesk software is used to setup a Graphical User Interface (GUI) that allows the real-time signals transmitted over the CAN bus and the estimated SOC/SOH signals to be monitored. The primary tasks associated with setting up the GUI involves

   - *Add Platform/Device*: Selecting the MABX ds1401 (corresponding to the dSPACE AutoMicroBox-II processor).
   - *Select Variable Description*: Assigning the previously generated *.sdf file corresponding to the estimation algorithm.

   The variables/parameters of the model in real-time are accessible from the *Variables Control bar* in the ControlDesk software. The GUI is populated with plotters to monitor the desired signals, such as the CAN signals (cell voltage, current, CAN communication status), and the estimated signals (SOC, SOH). When the application starts, the signals that are being monitored on the plotters are recorded, which is easily importable

to MATLAB and available for analysis.

References

Allam, A., Onori, S., 2018. An interconnected observer for concurrent estimation of bulk and surface concentration in the cathode and anode of a lithium-ion battery. IEEE Trans. on Ind. Electronics 65, 7311–7321.

Allam, A., Onori, S., 2020. Online capacity estimation for lithium-ion battery cells via an electrochemical model-based adaptive interconnected observer. IEEE Transactions on Control Systems Technology .

Di Domenico, D., Stefanopoulou, A., Fiengo, G., 2010. Lithium-ion battery state of charge and critical surface charge estimation using an electrochemical model-based extended kalman filter. Journal of dynamic systems, measurement, and control 132, 061302.

Edouard, C., Petit, M., Forgez, C., Bernard, J., Revel, R., 2016. Parameter sensitivity analysis of a simplified electrochemical and thermal model for li-ion batteries aging. J. of Power Sources , 482–494.

Prada, E., Di Domenico, D., Creff, Y., Bernard, J., Sauvant-Moynot, V., Huet, F., 2013. A simplified electrochemical and thermal aging model of lifepo4-graphite li-ion batteries: power and capacity fade simulations. Journal of The Electrochemical Society 160, A616.

Ramadass, P., Haran, B., White, R., Popov, B., 2003. Mathematical modeling of the capacity fade of li-ion cells. Journal of Power Sources 123, 230–240.
